# Sinonasal extraosseous Ewing’s sarcoma of the nasal cavity with EWSR1: FLI1 fusion – a rare case report from Nepal

**DOI:** 10.1097/MS9.0000000000004172

**Published:** 2025-10-20

**Authors:** Dipesh Kumar Singh, Isha Dhakal, Sanskriti Ghimire, Nitesh Pandit, Rajeev Sharma, Soniya Dulal

**Affiliations:** B.P. Koirala Institute of Health Sciences, Dharan, Nepal

**Keywords:** epistaxis, EWSR1::FLI1 fusion, nasal obstruction, sinonasal Ewing sarcoma

## Abstract

**Introduction:**

Ewing’s sarcoma is a rare, aggressive malignancy primarily originating in bones. Sinonasal extraosseous Ewing sarcoma (EES) displays morphologic heterogeneity that often correlates with underlying genotypes, most commonly the EWSR1::FLI1 fusion. EES, a soft tissue variant, constitutes less than 15% of all Ewing’s sarcoma cases and is exceptionally rare in the nasal cavity. Nasal EES presents with nonspecific symptoms such as nasal obstruction or bleeding, often leading to misdiagnosis and delayed treatment. Timely diagnosis through histopathological, immunohistochemical, and molecular testing is essential for initiating appropriate therapy and improving prognosis.

**Case Presentation:**

A 30-year-old female from Nepal presented with a 2-month history of a progressively enlarging mass in her left nasal cavity, accompanied by intermittent epistaxis and nasal obstruction. Physical examination revealed a firm, non-tender mass occupying the left nasal passage. Imaging showed a soft tissue lesion without bony erosion. Biopsy followed by histopathological analysis confirmed a diagnosis of sinonasal EES with demonstration of the EWSR1::FLI1 fusion by fluorescence *in situ* hybridization (FISH), showing characteristic small round blue cells. Immunohistochemistry was positive for CD99 and showed a Ki-67 index of 15–20%, indicating moderate proliferative activity. The patient received the first cycle of the chemotherapy regimen of vincristine, doxorubicin, cyclophosphamide, ifosfamide, and etoposide (VAC/IE) and was discharged in a stable condition.

**Discussion:**

Nasal EES is exceedingly rare, often mimicking benign lesions, which contributes to diagnostic delays. While CD99 immunoreactivity supports the diagnosis, its lack of specificity mandates molecular confirmation. Comprehensive differential diagnosis, including exclusion of other CD99-positive tumors, is critical. Multimodal treatment, including chemotherapy, surgery, and radiotherapy, is the standard approach. Early intervention is critical due to the tumor’s aggressive nature.

**Conclusion:**

This case emphasizes the need to consider EES in the differential diagnosis of nasal masses and highlights the necessity of molecular testing for EWSR1 rearrangements to confirm the diagnosis and guide therapy. Increased awareness and reporting are vital to enhancing diagnosis and management of this rare entity.

## Introduction

Ewing’s sarcoma is a highly malignant small round cell tumor of neuroectodermal origin, most commonly arising in the bones of children and young adults^[1]^. However, extraosseous Ewing’s sarcoma (EES) is a rare subset that arises in soft tissues without bone involvement^[2]^. The nasal cavity is an exceptionally uncommon site for EES, making early diagnosis challenging due to its resemblance to benign conditions such as nasal polyps, chronic sinusitis, or vascular tumors. Globally, Ewing’s sarcoma accounts for approximately 1–2% of all childhood cancers, with an annual incidence of 1.5 cases per million individuals^[3]^. Extraosseous variants are even rarer, with limited epidemiological data available. In Nepal, the incidence of Ewing’s sarcoma is underreported, but studies suggest it constitutes around 2–7% of primary bone malignancies, with extraosseous cases being exceedingly uncommon^[4]^. The World Health Organization (WHO) classifies Ewing’s sarcoma under the Ewing’s sarcoma family of tumors (ESFT), which includes classical osseous Ewing’s sarcoma, EES, and peripheral primitive neuroectodermal tumors (pPNET)^[5]^. These tumors share similar histological features but differ in clinical presentation and anatomical location. Diagnosing EES requires a combination of clinical suspicion, imaging, histopathology, immunohistochemistry, and molecular testing to detect EWSR1 gene rearrangements, with CD99 positivity being a useful but nonspecific marker^[5]^.

**HIGHLIGHTS**

- Rarity of nasal cavity extraosseous Ewing’s sarcoma (EES): limited global and Nepalese data exist on EES occurring in the nasal cavity.
- Diagnostic challenges: due to its nonspecific presentation, nasal EES is often misdiagnosed as benign tumors or chronic sinusitis.
- Unclear treatment protocols: while VAC chemotherapy is standard for osseous Ewing’s sarcoma, its long-term efficacy for nasal EES remains unproven.
- Prognosis uncertainty: there is insufficient data on survival rates, recurrence patterns, and treatment response in nasal EES.
- Lack of treatment guidelines: no consensus exists on the role of surgery and radiotherapy in managing nasal EES.

Treatment protocols for skeletal Ewing’s sarcoma, including multimodal chemotherapy with vincristine, doxorubicin, cyclophosphamide, ifosfamide, and etoposide (VAC/IE), have been adapted for EES. However, due to the tumor’s unique location in the nasal cavity, the role of surgery and radiation therapy remains uncertain. Long-term outcomes for patients with nasal EES are still not well documented, making it imperative to report such cases.

This report presents a case of molecularly confirmed sinonasal EES in a young adult female who was diagnosed and treated in resource-constrained settings like Nepal, aiming to contribute to the limited literature and highlight the importance of early recognition and management.

## Methods

Study design: The study is a case report (prospective, unicenter, formal, consecutive, and clinical). The work has been reported in line with the CARE 2013 criteria^[6]^.The article is compliant with the TITAN guidelines 2025^[7]^.

## Case presentation

A 30-year-old female with no significant medical history presented with complaints of a gradually enlarging left-sided nasal mass and intermittent nasal bleeding over several months. There was no underlying chronic illness or relevant family history. She denied symptoms of fever, weight loss, or systemic illness. On clinical examination, a firm, non-tender, and ulcerated mass was observed in the left nasal cavity. Imaging studies, including CT and MRI, revealed a heterogeneously enhancing soft tissue mass without bony destruction. Histopathology showed sheets of uniform small round blue cells with scant cytoplasm and fine chromatin. Immunohistochemistry was positive for CD99 (diffuse membranous) and NKX2.2, with negative staining for cytokeratin, desmin, and lymphoid markers, effectively excluding other small round cell tumors. FISH analysis demonstrated EWSR1 rearrangement, confirming EWSR1::FLI1 fusion.

The patient was admitted for chemotherapy initiation and received the first cycle of the VAC/IE regimen. She tolerated treatment well and was monitored for myelosuppression, mucositis, and nephrotoxicity. Her vital signs remained stable, and she was discharged in good condition with plans for continued chemotherapy cycles. Definitive management included ongoing multi-agent chemotherapy with consideration of adjuvant radiotherapy depending on response.

## Discussion

Ewing’s sarcoma is an aggressive malignancy, typically arising from primitive neuroectodermal cells. While its skeletal form is well documented, the extraosseous variant is rare, particularly in the nasal cavity. The exact pathogenesis of nasal EES remains unclear, but it is hypothesized to originate from neural crest-derived mesenchymal cells^[8]^. Due to its nonspecific presentation, nasal EES is frequently misdiagnosed as a benign tumor, leading to diagnostic delays. Given the non-specificity of CD99, comprehensive immunohistochemistry and molecular confirmation of EWSR1 rearrangements are mandatory to avoid misdiagnosis, as recommended in recent literature. The standard treatment for Ewing’s sarcoma involves multi-agent chemotherapy, with the VAC/IE regimen being widely used due to its effectiveness^[9]^. Surgical excision is often limited due to anatomical constraints, making chemotherapy the primary treatment modality. Radiotherapy may be beneficial in cases of incomplete remission.

A review of global literature indicates that nasal EES accounts for less than 1% of all Ewing’s sarcoma cases^[3]^. Updated reviews emphasize genotype-phenotype correlations, particularly the EWSR1::FLI1 fusion, which usually drives classic morphology, whereas EWSR1::ERG or FUS::ERG fusions may produce more atypical features. Prognostic data for nasal EES remain scarce, but studies suggest that skeletal Ewing’s sarcoma has a 5-year survival rate of approximately 60–70% with aggressive treatment^[10]^. For EES, survival rates vary between 40% and 60%, depending on tumor location and treatment response. Small case series suggest a relatively poorer prognosis for nasal EES, attributed to delayed diagnosis and challenges in achieving complete surgical resection.

Managing EES in Nepal presents unique challenges, including limited access to advanced diagnostic tools such as genetic testing for EWSR1 translocation, delays in referrals due to low clinical suspicion, and financial constraints that restrict access to multimodal therapy. In resource-limited settings, chemotherapy remains the primary treatment approach, with the VAC/IE regimen demonstrating moderate effectiveness. Increasing awareness among healthcare providers about rare malignancies like nasal EES can facilitate earlier diagnosis and intervention, potentially improving patient outcomes.

## Conclusion

Sinonasal EES with EWSR1::FLI1 fusion is an exceptionally rare malignancy that poses significant diagnostic and therapeutic challenges. This case highlights the importance of early clinical suspicion, histopathological confirmation, molecular testing, and prompt chemotherapy initiation. Given the limited literature on nasal EES, reporting such cases is crucial for improving our understanding of its biological behavior, treatment response, and long-term prognosis. Further research is necessary to establish standardized treatment guidelines for this rare entity. Heightened clinical awareness and improved diagnostic capabilities can lead to earlier detection and better survival outcomes for patients with nasal EES, particularly in resource-constrained settings like Nepal.

## Data Availability

The data that support the findings of this study are available from the corresponding author upon reasonable request.

## References

[1] Chin EW Abu-Bakar AZ Hitam S. Primary extraosseous ewing sarcoma of the maxillary sinus in an adult-a rare case report. Iran J Otorhinolaryngol 2019;31:391–97. 31857985 10.22038/ijorl.2019.35555.2173 PMC6914322

[2] Sethi P Singh A Srinivas BH. Practical approach in management of extraosseous ewing’s sarcoma of head and neck: a case series and review of literature. Gulf J Oncolog 2022;1:79–88. 35695350

[3] Arora RS Alston RD Eden TO. The contrasting age-incidence patterns of bone tumours in teenagers and young adults: implications for aetiology. Int J Cancer 2012;131:1678–85. 22174047 10.1002/ijc.27402

[4] Yeshvanth SK Ninan K Bhandary SK. Rare case of extraskeletal Ewings sarcoma of the sinonasal tract. J Cancer Res Ther 2012;8:142–44. 22531536 10.4103/0973-1482.95197

[5] Galyfos G Karantzikos GA Kavouras N. Extraosseous ewing sarcoma: diagnosis, prognosis and optimal management. Indian J Surg 2016;78:49–53. 27186040 10.1007/s12262-015-1399-0 PMC4848231

[6] Riley DS Barber MS Kienle GS. CARE guidelines for case reports: explanation and elaboration document. J Clin Epidemiol. 2017;89:218–35. 28529185 10.1016/j.jclinepi.2017.04.026

[7] Agha RA Mathew G Rashid R. TITAN Group. Transparency In The reporting of Artificial INtelligence – the TITAN guideline. Premier J Sci. 2025;10:100082.

[8] Granowetter L. Ewing’s sarcoma. CurrOpinOncol 1991;3:684–88. 1932228

[9] Anderton J Moroz V Marec-Bérard P. International randomised controlled trial for the treatment of newly diagnosed EWING sarcoma family of tumours – EURO EWING 2012 protocol. Trials 2020;21:96. 31952545 10.1186/s13063-019-4026-8 PMC6969439

[10] Zhan H Mo F Zhu M. A SEER-based nomogram accurately predicts prognosis in Ewing’s sarcoma. Sci Rep 2021;11:22723. 34811459 10.1038/s41598-021-02134-0 PMC8608824

